# Microenvironments and Cellular Characteristics in the Micro Tumor Cords of Malignant Solid Tumors

**DOI:** 10.3390/ijms131113949

**Published:** 2012-10-29

**Authors:** Chan Joo Yeom, Yoko Goto, Yuxi Zhu, Masahiro Hiraoka, Hiroshi Harada

**Affiliations:** 1 Group of Radiation and Tumor Biology, Career-Path Promotion Unit for Young Life Scientists, Kyoto University, Yoshida Konoe-cho, Sakyo-ku, Kyoto 606-8501, Japan; E-Mails: cjyeom@kuhp.kyoto-u.ac.jp (C.J.Y.); ygoto@kuhp.kyoto-u.ac.jp (Y.G.); zhuyuxi@kuhp.kyoto-u.ac.jp (Y.Z.); 2 Department of Radiation Oncology and Image-applied Therapy, Kyoto University Graduate School of Medicine, 54 Shogoin Kawahara-cho, Sakyo-ku, Kyoto 606-8507, Japan; E-Mail: hiraok@kuhp.kyoto-u.ac.jp; 3 Department of Oncology, The First Affiliated Hospital of Chongqing Medical University, No.1 Friendship Road, Yuanjiagang, Yuzhong District, Chongqing 400016, China

**Keywords:** tumor microenvironment, hypoxia, glucose availability, hypoxia-inducible factor 1 (HIF-1), radioresistance, chemoresistance, metabolism

## Abstract

Because of the accelerated proliferation of cancer cells and the limited distance that molecular oxygen can diffuse from functional tumor blood vessels, there appears to be a unique histology in malignant solid tumors, conglomerates of micro tumor cords. A functional blood vessel exists at the center of each tumor cord and is sequentially surrounded by well-oxygenated, oxygen-insufficient, and oxygen-depleted cancer cells in the shape of baumkuchen (layered). Cancer cells, by inducing the expression of various genes, adapt to the highly heterogeneous microenvironments in each layer. Accumulated evidence has suggested that not only tumor microenvironments but also cellular adaptive responses to them, influence the radioresistance of cancer cells. However, precisely how these factors affect one another and eventually influence the therapeutic effect of radiation therapy remains to be elucidated. Here, based on recent basic and clinical cancer research, we deduced extrinsic (oxygen concentration, glucose concentration, pH *etc.*) and intrinsic (transcriptional activity of hypoxia-inducible factor 1, metabolic pathways, cell cycle status, proliferative activity *etc.*) parameters in each layer of a tumor cord. In addition, we reviewed the latest information about the molecular mechanism linking these factors with both tumor radioresistance and tumor recurrence after radiation therapy.

## 1. Introduction

**Figure 1:**
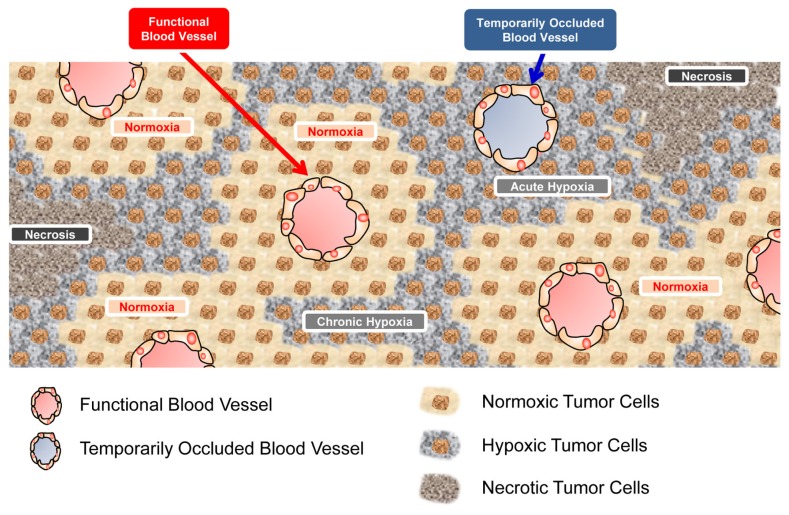
Schematic diagram of micro tumor cords. Because of the limited distance that molecular oxygen can diffuse from functional blood vessels, cancer cells can obtain oxygen only in close proximity to functional vessels (normoxic regions). Cancer cells approximately 70–100 μm from blood vessels cannot obtain oxygen (hypoxic regions). Cancer cells even farther away become necrotic. Because blood vessels in malignant solid tumors are immature and highly tortuous, they are sometimes occluded temporarily. Then, cancer cells surrounding dysfunctional vessels are known to be exposed to acute hypoxia.

Malignant solid tumors grow rapidly with an insufficient vasculature, leading to an imbalance between oxygen-consumption in and oxygen-supply to the cancer cells [1,2]. Such disequilibrium and the limited diffusion of molecular oxygen from tumor blood vessels cause a tumor-specific microenvironment, hypoxia [1,2]. Most malignant tumors consequently grow as an assembly of micro tumor cords, in each of which a blood vessel is surrounded by well-oxygenated (normoxic), oxygen-insufficient (hypoxic), and finally oxygen-depleted (anoxic/necrotic) cancer cells (Figure 1) [1,2].

Cancer cells adapt to low-oxygen conditions by inducing the expression of a transcription factor, hypoxia-inducible factor 1 (HIF-1). HIF-1 is a heterodimer composed of HIF-1α and HIF-1β subunits [3]. While HIF-1β is constitutively expressed, the expression and transactivation activity of the HIF-1α subunit are known to be induced by hypoxic stimuli [3]. The oxygen-dependent degradation (ODD) domain of HIF-1α is hydroxylated by prolyl hydroxylases in an oxygen-dependent manner [4,5]. The modification triggers the ubiquitination of the subunit by a pVHL-containing E3 ubiquitin ligase and subsequent proteolysis by the 26S proteasome complex [4,5]. Conversely, HIF-1α becomes relatively stable under hypoxic conditions because the depletion of oxygen directly inactivates prolyl hydroxylases [5]. Then, the stabilized HIF-1α interacts with HIF-1β to form an active heterodimer, HIF-1. HIF-1 binds to its cognate enhancer sequence, the hypoxia-response element (HRE), and induces the expression of various genes related to the adaptation of cellular metabolism to hypoxia (the switch from oxidative to anoxic respiration: metabolic reprogramming) [6], escape from hypoxia (invasion and metastasis of cancer cells) [7,8], and improvement of hypoxia (angiogenesis) [9,10].

Tumor hypoxia and HIF-1 have received considerable attention especially in radiation oncology because they affect tumor radioresistance [2,11–13]. Ionizing radiation is known to produce free radicals by interacting primarily with the water in a cell and damage DNA regardless of the presence or absence of molecular oxygen. However, molecular oxygen functions in the oxidization of the damaged DNA, converting the damage into a form that is more difficult for the cell to repair and therefore more lethal. Thus, cells suffer from more DNA damage under normoxic conditions and less damage under hypoxic conditions [14]. In addition to such a radiation-chemical mechanism, hypoxia is known to increase the radioresistance of malignant solid tumors also through HIF-1-mediated biological mechanisms [15–20]. Actually, there is clinical evidence that the size of hypoxic regions and the level of HIF-1α in tumors correlate with a poor prognosis after radiation therapy [21–24].

It has been suggested through extensive *in vitro* studies that hypoxia- and/or HIF-1-associated factors, such as acidity, cell cycle arrest, proliferative potential, and cellular metabolic pathways *etc.*, potentially affect the radioresistance of cancer cells [12,25,26]. Here, we summarize how these factors affect one another in each layer of the micro tumor cord so as to better understand the mechanisms behind tumor radioresistance and tumor recurrence after radiation therapy. In addition, we summarize microenvironments of several layers in micro tumor cords mainly based on our recent findings through *in vitro*, *in vivo*, and *ex vivo* experiments. The information is quite important; however, one should carefully consider the generality of the summarized information because solid tumors can be profoundly variable among different types and even among the same type.

## 2. Tumor Microenvironments and Cellular Characteristics in a Micro Tumor Cord

### 2.1. HIF-1α Expression in a Micro Tumor Cord

#### 2.1.1. HIF-1α-Positive and HIF-1α-Negative Populations in Hypoxic Regions

The expression of HIF-1α is known to dramatically increase as the oxygen concentration decreases *in vitro*[27]. For example, we confirmed that the expression of the HIF-1α protein and transcriptional activity of HIF-1 was induced at 1% and 0.02% oxygen, respectively, in a cervical cancer cell line, HeLa [28]. (Some papers reported slight expression at 3% but others reported no expression at 3% oxygen.) These results led us to postulate that HIF-1α expression would increase farther from the blood vessel in a micro tumor cord. Moreover, we had simply thought that cancer cells stained with pimonidazole in perinecrotic regions would show high HIF-1α expression. However, recent immunohistochemical analyses revealed that this is not necessarily true. Immunostaining with both pimonidazole and an antibody against HIF-1α or a downstream gene of HIF-1 unveiled that the hypoxic layer is mainly composed of two layers; a HIF-1α-positive/pimonidazole-negative layer and a pimonidazole-positive/HIF-1α-negative layer [1,28–31] (Table 1). The HIF1α-positive/pimonidazole-negative layer is located closer to tumor blood vessels than the pimonidazole-positive/HIF-1α-negative layer. The reduced expression of HIF-1α in perinecrotic regions (border between necrosis and hypoxia) has been observed in various human tumors and in xenografts of SiHa cervical carcinoma, WiDr colon carcinoma, and M006 astrocytoma [30].

#### 2.1.2. Mechanism behind the Decrease in HIF-1α Expression in Perinecrotic Regions

So, why is the expression of HIF-1α decreased in perinecrotic regions (pimonidazole-positive/HIF-1α-negative layer), although cancer cells should be exposed to lower O_2_ conditions there compared to the HIF1α-positive/pimonidazole-negative regions because of the distance from blood vessels? Our group successfully identified “low glucose-availability” as an important factor [31]. Experiments *in vitro* demonstrated that a decrease in the glucose concentration reduced the level of HIF-1α protein, transcriptional activity of HIF-1, and expression of downstream genes even under hypoxic conditions [28,31]. Immunohistochemical analyses *in vivo* showed that, when tumor-bearing mice were continuously administered glucagon, which increases blood glucose concentrations and so improves glucose-availability in the pimonidazole-positive hypoxic regions, HIF-1α expression was dramatically induced there [31]. These results clearly show the involvement of decreased glucose-availability in the reduced HIF-1α expression (see Section 2.3 for details about the glucose concentration in each layer of a micro tumor cord). Precisely how glucose-availability influences HIF-1α expression remains to be elucidated.

### 2.2. Oxygen Concentration in a Micro Tumor Cord

#### 2.2.1. Oxygen Concentrations in the Pimonidazole-positive/HIF-1α-negative Layer

A number of methods have been developed to assess the concentration of oxygen in malignant solid tumors, including the direct measurement of partial oxygen pressure (pO_2_) by using oxygen microelectrodes, immunohistochemical analysis, and positron emission tomography (PET) using hypoxic markers (e.g., pimonidazole hydrochloride for immunohistochemistry, and misonidazole [32], and ^62^Cu-ATSM for PET) [33–36].

In the late 1980’s, a clinically applicable standardized procedure was established by using a computerized polarographic needle electrode system [36], which enables the determination of oxygenation in accessible primary tumors, locally recurrent tumors, and metastatic lesions. Using this technique, Vaupel *et al.* found the overall median pO_2_ in cancers of the uterine cervix, head and neck, and breast to be approximately 10 mmHg, with the overall hypoxic fraction (pO_2_ value < or = 2.5 mmHg) being approximately 25% [36]. In contrast, pO_2_ values lower than 12.5 mmHg were not observed in normal tissues in breast cancer patients [37]. Although these results gave a very important, comprehensive, and panoramic view of tumor hypoxia, this approach is not satisfactory for obtaining microregional information.

In order to elucidate pO_2_ values at a microregional level, immunohistochemical analyses using several hypoxic markers have been developed. Nitroimidazole derivatives, such as pimonidazole hydrochloride and EF5, are representative markers [35,38]. Their unique characteristics of being specifically reduced under hypoxic conditions and forming a covalent bond with thiol groups of arbitrary proteins in cells enables the detection of hypoxic regions. Cancer patients should be administered the marker, and then tumor tissues surgically excised and subjected to immunostaining with antibodies. Basic as well as clinical research has revealed that hypoxic cells detected by the markers are principally located 85–100 μm from perfusion-positive/functional blood vessels in malignant solid tumors [28,29]. Because the reduction of nitroimidazole-derivatives occurs at oxygen tensions of 10 mmHg or less (approximately 1.3%) [35,39] and because our *in vitro* study showed that cells were stained with pimonidazole at less than 1% oxygen and but not at 3% oxygen [28], cells detected with the nitroimidazole-derivatives would be under such oxygen-conditions (Table 2).

#### 2.2.2. Oxygen Concentrations in the HIF-1α-Positive/Pimonidazole-Negative Layer

Given the characteristics of both pimonidazole and HIF-1α, we are also able to estimate the range of oxygen concentrations in the HIF-1α-positive/pimonidazole-negative layer (Table 2). Cells in this layer have been thought to be under relatively mild hypoxia, higher than at least 1%, because, if they were under severer hypoxia, e.g., less than 1%, they should have been stained with pimonidazole [28,35]. On the other hand, this layer has been assumed to be under no higher than 3% oxygen because a series of *in vitro* studies confirmed that HIF-1α is expressed at less than approximately 3% oxygen. This notion is further supported by our recent study; a HIF-1-dependent enzymatic reaction was not induced when the oxygen concentration was 3% or higher [28].

#### 2.2.3. Oxygen Concentrations in Normoxic Regions

It is reasonable to assume that oxygen concentrations are higher in normoxic regions than hypoxic layers because of the proximity to the blood vessel in a tumor cord. However, it is difficult to calculate a value because the data regarding oxygen concentrations which can induce *in vitro* HIF-1α expression are controversial. Based on our own studies *in vitro*, HeLa cells express HIF-1α and show HIF-1 activity under 1% but not 3% hypoxic conditions. On the other hand, some research groups demonstrated HIF-1α expression at 3% oxygen. In addition, several papers estimated maximal oxygen tensions in solid tumors to be between 2% and 5% O_2_[40–43]. Therefore, all that can be deduced at this point is that oxygen concentrations in the normoxic layer are much higher than 1%, probably higher than 3%, and not more than 5% (Table 2).

#### 2.2.4. Acute/Intermittent/Cycling Hypoxia

In addition to oxygen diffusion-limited hypoxia (chronic hypoxia), there is also another form of tumor hypoxia characterized by fluctuating changes in pO_2_ as a result of the disorganized and highly tortuous tumor vascular. [44]. This complex feature of temporal instability in oxygen transport has classically been termed acute/intermittent/cycling hypoxia. Chaplin and Durand suggested that there was a dominant cycle time of 20–30 min for fluctuations in hypoxia, based on dye mismatch studies [45]. On the other hand, Brown suggested a slower variations of pO_2_ fluctuation, such as a 24 h interval [44]. The primary consequence of cycling hypoxia is an upregulation of HIF-1 activity to a level equivalent to that under chronic hypoxia. Martinive *et al*. found that repeated exposure of endothelial cells to hypoxia and reoxygenation can lead to much greater upregulation of HIF-1 than that achieved by prolonged exposure to hypoxia [46]. They suggested that cycling hypoxia may condition endothelial cells and tumor cells in such a way that they are more resistant to apoptosis and more prone to participate in tumor progression. In addition, Cairns *et al.* found that exposure of tumor-bearing mice to cycling (not chronic) hypoxia significantly increased the incidence of metastasis to the lungs [47].

#### 2.2.5. Oxygen Concentrations in a Micro Tumor Cord

Taken together, the biologically-estimated concentrations of oxygen in the three layers, normoxic cells, HIF-1α-positive/pimonidazole-negative hypoxic cells, and HIF-1α-negative/pimonidazole-positive hypoxic cells are >>1%, 1%–3%, and <1%, respectively (Table 2).

### 2.3. Glucose-Availability in a Micro Tumor Cord

Glucose is a simple sugar that primarily functions as an energy source providing adenosine triphosphate (ATP). The availability of glucose in a malignant solid tumor is one of the local regulators which affect the growth of individual cancer cells and the progression of whole tumors. Because of the limited distance that glucose as well as oxygen can diffuse from tumor blood vessels, cancer cells farther away from blood vessels cannot get enough glucose [31]. We revealed that the decrease of glucose-availability results in the suppression of HIF-1α expression in pimonidazole-positive/HIF-1α-negative regions in a tumor cord, as described above (See Section 2.1) [31]. So, how heterogeneous is glucose-availability/glucose-distribution in a malignant solid tumor? Walenta *et al.* conducted metabolic mapping in both tumor xenografts and clinical biopsies with quantitative bioluminescence and single photon imaging techniques [48]. They suggested a significant reduction in ATP and glucose especially in perinecrotic regions. Another group demonstrated the same possibility. When the glucose availability in perinecrotic regions of a micro tumor cord was increased through continuous administration of glucagon by using an osmotic pump, the expression of HIF-1α in these regions was significantly increased [31]. In addition, we revealed, by performing an *in vitro* experiment, that HIF-1α expression, HIF-1 activity, and the expression of HIF-1 downstream genes were suppressed when the glucose concentration in the culture medium was reduced to 0.45 g/L (2.49 mM) even under 1% and 0.02% oxygen conditions [28,31]. These results directly indicate that cancer cells in HIF-1α-positive/pimonidazole-negative regions should be supplied with enough glucose, at least higher than 0.45 g/L, for the expression of HIF-1α. On the other hand, cancer cells in perinecrotic/pimonidazole-positive regions are under low glucose conditions, less than 0.45 g/L (2.49 mM), resulting in a decreased level of HIF-1α even under hypoxic conditions (Table 3).

A relationship between the intra-tumor heterogeneity of glucose availability and tumor metastasis was also demonstrated by using clinical data on head and neck carcinomas [48]. There was a significant difference between the metastatic group and non-metastatic group with regard to the glucose content of primary lesions. Interestingly, the glucose level of the metastatic group was significantly lower. This study implies that glucose-availability in primary tumors might be associated with a poor prognosis. In addition to glucose-availability, the expression of a glucose transporter gene, which functions in glucose-uptake into cells, is recognized as an important prognostic factor.

### 2.4. *pH* in a Micro Tumor Cord

Several studies have shown that the extracellular pH in human tumors as well as experimental xenografts is low, around 6.0. Such a microenvironment is, at least in part, caused by CO_2_ and lactate produced by and secreted from cancer cells through accelerated glycolysis and lactic acid fermentation [6]. Because hypoxia is one of the most remarkable triggers for the acceleration [49], there is a possibility that intratumoral pH mapping is affected by the distance from functional tumor blood vessels. Cancer cells shift their metabolic pathway from mitochondrial oxidative phosphorylation to glycolysis and lactic acid fermentation so as to adjust their oxygen-demand to meet the limited oxygen-supply under hypoxic conditions [50,51]. HIF-1 has been reported to play important roles in the metabolic reprogramming through the following functions. First, HIF-1 upregulates glucose metabolism. HIF-1 induces the expression of genes involved in glucose uptake (such as GLUT1 and GLUT3) [52] and enzymatic breakdown of glucose to pyruvate (such as HK2) [53]. Furthermore, due to the increase of lactate dehydrogenase A (LDH-A) expression, HIF-1 facilitates lactic acid fermentation (further exchange of pyruvate to lactate) [6]. Second, HIF-1, by inducing the expression of pyruvate dehydrogenase kinase 1 (PDK1) and inactivating the pyruvate dehydrogenase complex (PDH), inhibits the conversion of pyruvate to acetyl-CoA and decreases the supply of a substrate to the tricarboxylic acid (TCA) cycle and the electron transport chain [54,55]. Third, HIF-1 is reported to act in the downregulation of mitochondrial function. Experiments using functional VHL-deficient renal cell carcinoma cell lines revealed that HIF-1 can reduce mitochondrial biogenesis by inducing the expression of MAX interactor 1 (MXI-1), which represses c-Myc transcriptional activity, and promoting MXI-1-independent but proteasome-dependent degradation of c-Myc. MXI-1 helps to inhibit the expression of genes associated with mitochondrial DNA replication [56–58]. On the other hand, HIF-1 is also known to induce the autophagy of mitochondria by inducing the expression of BNIP3 [59]. Another mechanism by which HIF-1 controls mitochondrial function is the regulation of the expression of the COX4 subunits by activating transcription of the genes encoding COX4-2 and LON, a mitochondrial protease that is required for degradation of COX4-1 [60].

In addition to the HIF-1-mediated metabolic reprogramming, HIF-1-dependent expression of carbonic anhydrase 9 (CA9) on the tumor cell surface also contributes to extracellular acidification by hydrating CO_2_ to HCO^3−^ and H^+^[61]. The accumulated lactate within tumor cells can be co-transported out of the cell with H^+^ via monocarboxylate transporters (MCTs), resulting in a decrease of extracellular pH (Table 4) [62].

Based on the notion of HIF-1-mediated metabolic reprogramming described above, only HIF-1α-positive hypoxic regions would be under acidic conditions in a micro tumor cord and normoxic regions would be under neutral pH. However, it does not seem to be that simple. It is now evident that HIF-1α expression and HIF-1 activity are regulated by various factors even under normoxic conditions; e.g., through the activation of oncogenes or loss of tumor suppressor genes [63], by increased levels of metabolites such, as succinate and furmarate, by reactive oxygen species (ROS) [64], and the products of glycolysis, such as lactate and pyruvate *etc.*[65]. Moreover, a number of oncogenes and tumor suppressor genes, such as AMP-activated protein kinase (AMPK), NF-kB, myc, epidermal growth factor (EGF), insulin-like growth factor I, phosphoinositol 3 kinase (PI3K), mTOR, and Kirsten rat sarcoma viral oncogene homolog (KRAS), have been directly associated with the metabolic reprogramming [66,67]. Also, c-Myc has been shown to be linked to the regulation of glycolysis in aerobic cells through the direct activation of LDHA and almost all glycolytic genes [68,69], and mutated Ras is known to enhance glycolysis, partly by increasing the stability of c-Myc [70]. From these points of view, metabolic reprogramming and the resultant acidic conditions should be generated not only in hypoxic regions in a HIF-1-dependent manner but also in normoxic regions. In fact, Warburg *et al.* reported in their pioneering studies in the 1920s that tumor tissues metabolize more glucose to lactate than normal tissues even in the presence of enough O_2_, a phenomenon known as the Warburg effect [71]. If so, pH in normoxic regions may be dependent on the extent of the Warburg effect (Table 4). In order to spatio-temporally understand the pH map in a conglomerate of micro tumor cords, it is critical to elucidate how tumor-specific microenvironments and mutations of oncogenes and/or tumor-suppressor genes influence the Warburg effect.

Intratumoral pH has been indirectly measured by using fluorescence ratio imaging microscopy. Dellian *et al.* reported that steep interstitial pH gradients exist between tumor blood vessels; the pH decreased by an average of 0.10 pH units over a distance of 40 microns away from the blood vessel wall, and by 0.33 pH units over a 70 microns distance [72]. They additionally reported that the maximum pH drop, defined as the pH difference between the inter-vessel midpoint and the vessel wall, was positively correlated with the inter-vessel distance [72]. Moreover, Helmlinger *et al.* found a plateau phase of the mean pH, the pH = 6.91 100–170 μm from blood vessels. Furthermore, the pH decreased further to a second plateau at 6.70 in anoxic/necrotic regions [41].

### 2.5. Cell Cycle Status and Proliferative Activity of Cells in a Micro Tumor Cord

A fundamental biological process that is dramatically influenced by oxygen-availability is cell proliferation. In many cell types, hypoxia suppresses cell proliferation. Because cancer cells away from functional blood vessels receive minimum amounts of nutrients and oxygen from the circulation, they show the lowest proliferative index. In 1997, a quantitative comparison of immunostaining for hypoxia and proliferation indicated an inverse relationship between pimonidazole binding and proliferation markers in human squamous cell carcinomas [73]. Koshiji *et al.* demonstrated that acute stabilization of HIF-1α and subsequent activation of HIF-1 induce cell cycle arrest under hypoxia (1% O_2_). HIF-1 inhibits the function of c-Myc through physical binding to its *N*-terminal region [74]. Over 40% of human cancers exhibit overexpressed c-Myc, regulating the aberrant tumor cell proliferation and metabolism. c-Myc controls the G_1_/S transition by associating with MAX. This heterodimer promotes the expression of genes such as cyclin D2 (CCND2), E2F1 and ornithine decarboxylase 1 (ODC1). Simultaneously, c-Myc inhibits the expression of CDKN1A and CDKN1B encoding the cyclin-dependent kinase inhibitors (CDKIs) p21 and p27, respectively[75]. Through multiple mechanisms, HIF-1 effectively inhibits c-Myc-dependent cell proliferation, and then seems to induce cell cycle arrest at G_1_. So, G_1_ arrest would be induced in the HIF-1α-positive/pimonidazole-negative layer of a micro tumor cord (Table 5) [56,76], however, it is also true that a proliferation-dependent enzymatic reaction could be induced in this layer, meaning that HIF-1α-positive cells are still proliferative [28]. From this point of view, the proliferative potential of HIF-1α-positive/pimonidazole-negative cells still remains controversial (Table 5).

In contrast, the proliferative activity of cancer cells seems to be relatively attenuated in pimonidazole-positive/HIF-1α-negative layers. We recently revealed that a low glucose-mediated decrease in HIF-1α expression results in the suppression of p27 expression, leading to release from cell cycle arrest and subsequent transition from the G_1_ to S phase [31]. As a result, the proportion of cancer cells in S-phase significantly increases in pimonidazole-positive/HIF-1α-negative layers (although a substantial number of cells seem to remain in G_1_/G_0_). We additionally found that, after the progression of a certain percentage of cells into S-phase, the cell cycle remains at S-phase without efficient DNA synthesis probably because of the depletion of glucose and oxygen [31].

Liu *et al.* investigated biological functions such as the cell cycle and proliferation in lung cancer cells treated with cycling hypoxia *in vitro*. They found that repeated exposure of A549 and H446 lung cancer cells to 0.1% O_2_ hypoxia and 20% O_2_ normoxia for 20 cycles resulted in higher proliferation rates than in the parental cell lines based on their MTT assay. They also showed that the S-phase population of A549 and H446 cells exposed to cycling hypoxia increased. These results suggest that cycling hypoxia could promote cellular proliferation [77].

## 3. Radio-Resistance of Cancer Cells in a Micro Tumor Cord

### 3.1. Mechanism behind Radioresistance of Perinecrotic Tumor Cells and Tumor Recurrence

Some intracellular and extracellular factors, e.g., HIF-1 activity, cell cycle status, and oxygen-availability, have been suggested to influence the radioresistance of cancer cells. Clinical research has demonstrated that the overall survival rate of cancer patients with high intratumoral HIF-1 activity was significantly worse than that of patients with low HIF-1 activity after radiation therapy [2,11–13]. Moreover, basic research has demonstrated that tumor growth after radiation therapy was significantly delayed when an adenovirus expressing siRNA targeting HIF-1α was administered [78]. As for cell cycle status, the radioresistance of cells is significantly higher in S-phase than G_1_ phase [26] because there is a sister chromatid in S-phase, which is needed for the repair of DNA lesions through homologous recombination. Also, cells are approximately three times more radioresistant in the absence of oxygen, because oxygen-depletion disturbs the accumulation of reactive and cytotoxic species by ionizing radiation [79]. Moreover, DNA radicals, which are barely produced under hypoxia through the ionization of DNA, can be reduced by SH group-containing biological materials, decreasing DNA damage [79].

We recently analyzed mechanistic and spatio-temporal relationships among these intrinsic and extrinsic factors [31]. We unveiled that, because of the insufficient delivery of glucose, most cancer cells in pimonidazole-positive regions are HIF-1α-negative in HeLa xenografts as described above. The resultant downregulation of HIF-1 activity leads to a transition from G_1_ to S phase because of the suppression of p27 expression. The G_1_-S transition actually induces resistance of cells to radiation-induced DNA damage (Table 6).

Then, we next examined whether cancer cells in the pimonidazole-positive/HIF-1α-negative layer actually survive radiation therapy and directly cause tumor recurrence [28]. We established a strategy to tag these cells with luciferase bioluminescence at a precise window in time by using a hypoxia-dependent Cre-ER^T2^/loxP system. By performing an optical cell tracking experiment, we identified these cells as an important population which predominantly survive radiation and directly cause tumor recurrence. This conclusion may help to explain why earlier studies, which aim to validate intrinsic markers of hypoxia such as Glut-1, CA9 and HIF-1, have showed poor correlations with the results obtained by using bio-reductive hypoxic probes, such as 2-nitroimadazole derivatives, and oxygen electrode measurements.

However, we also realized that we should not ignore the importance of HIF-1 as a therapeutic target in radiation therapy [80]. This is because there is substantial evidence that HIF-1 becomes active in perinecrotic regions after radiation treatment, inducing the expression of vascular endothelial cell growth factor, which potentially protects tumor blood vessels from the cytotoxic effects of radiation, assures the delivery of oxygen and nutrients to cancer cells, and eventually accelerates tumor growth after the radiation treatment [15,17–19,81]. Moreover, the radiation-induced activation of HIF-1 triggers translocation of the radio-surviving cells from perinecrotic regions toward the tumor vasculature and leads to tumor recurrence[28].

It might be also true that acidic conditions would positively influence radioresistant characteristics of cancer cells at least indirectly, although detailed mechanisms of action have not yet been elucidated. Cancer cells are exposed to acidic conditions under hypoxia because of the enhanced glycolysis as mentioned above (See Section 2.4). It is reasonable to assume that cancer cells, which adapt to such severe acidic conditions, should acquire radioresistant characteristics, too. If so, cancer cells which show high levels of aerobic glycolysis because of mutations of oncogenes or tumor suppressor genes might be also radioresistant even in normoxic layers in a micro tumor cord.

### 3.2. Strategies to Overcome Radioresistance of Cancer Cells

Taken together, the parameters most important for radiation therapy would be the extent of pimonidazole-positive hypoxia (absolute hypoxia) before radiation and the extent of HIF-1α-positive regions and intratumoral HIF-1 activity after radiation. Moreover, on the basis of these findings, the therapeutic effect of the triple combination of radiation therapy, a hypoxia cytotoxin (for the killing of pimonidazole-positive cells; tirapazamine *etc.*), and a HIF-1 inhibitor (for the suppression of radiation-induced activation of HIF-1; topotecan, doxorubicin, HSP90 inhibitor, and YC-1 *etc.*) should be examined. Also, we should establish a strategy to visualize the radioresistant populations and deliver booster doses of radiation against them. Moreover, a combination of radiation therapy with hyperbaric oxygen treatment, which enables the oxygenation of hypoxic areas, has drawn considerable attention recently. Hypoxic cell radiosensitizer, such as nimorazole, may also overcome the problems related to hypoxia.

Based on the information about the importance of absolute hypoxia in tumor radioresistance, we can understand how fractionated radiation therapy functions in overcoming the radioresistance. Ionizing radiation efficiently kills cancer cells in proximal regions of tumor blood vessels because of the presence of molecular oxygen. Then, oxygen consumption dramatically decreases at this point, and consequently, oxygen gets to diffuse to more distal regions. This phenomenon, known as reoxygenation of hypoxic cells, increases the therapeutic effect of subsequent fractionated radiation against the ex-hypoxic/reoxygenated cells. However, there is also a suggestion that the concept of a hypoxic fraction is less meaningful when pertaining to fractionated radiation therapy [82]. Wouters and Brown reported that the impact of full reoxygenation between fractions is much smaller than previously realized [82]. They, at the same time, reported that the response of entire tumors to radiation therapy is highly dependent upon the cells at oxygen levels intermediate between fully oxygenated and hypoxic (0.5–20 mmHg) [82]. Consequently, one should not neglect the importance of both absolute and intermediate hypoxia in fractionated radiation therapy.

## 4. Perspective

To fully comprehend how cancer cells acquire radioresistance, it is important to understand the spatio-temporal relationships among the various extrinsic and intrinsic factors in a highly heterogeneous malignant solid tumor composed of multiple micro tumor cords. Accumulated biological data have actually contributed to answering this question, but there still remain some important problems. First, most of the information documented here was obtained through basic research using experimental tumor xenografts whose growth rate is far different from that of human tumors; therefore, we have to examine whether the parameters discussed here can be applied to real human tumors. Second, it is not clear how and why HIF-1α expression, which is the most influential factor affecting intratumoral pH, cell cycle status, oxygen-consumption, and moreover tumor radioresistance *etc.*, is suppressed under hypoxic conditions when the glucose concentration is low [31]. Third, it is also unknown how the cell cycle can be arrested at S phase after it progresses from G_1_ to S phase under low glucose and hypoxic conditions [31]. Answers to these questions should contribute to the development of novel strategies to overcome tumor radioresistance.

## Figures and Tables

**Table 1:** Hypoxia-inducible factor 1α (HIF-1α) Expression and the Pimonidazole Reaction in Each Layer of a Micro Tumor Cord.

	Normoxic regions	Hypoxic regions		Necrotic regions

		HIF-1α^+^/pimo^−^	Pimo^+^/HIF-1α^−^	
Distance from a Blood Vessel	Close	Far (about 70–85 μm)	Farther (about 85–100 μm)	Extremely Far (<<100 μm)
HIF-1α Expression	−	+	−	−
Pimo Staining	−	−	+	−

**Table 2:** Oxygen Concentrations in Each Layer of a Micro Tumor Cord.

	Normoxic regions	Hypoxic regions		Necrotic regions

		HIF-1α^+^/Pimo^−^	Pimo^+^/HIF-1α^−^	
Oxygen Concentrations	>>1% (or > about 3%) (<5%)	1%–3%	<1%	<<1%

**Table 3:** Glucose Concentrations in Each Layer of a Micro Tumor Cord.

	Normoxic regions	Hypoxic regions		Necrotic regions

		HIF-1α^+^/Pimo^−^	Pimo^+^/HIF-1α^−^	
Glucose Concentrations	Extremely High (>>2.5 mM)	High (>2.5 mM)	Low (<2.5 mM)	Extremely Low (<<2.5 mM)

**Table 4:** The pH in Each Layer of a Micro Tumor Cord.

	Normoxic regions	Hypoxic regions		Necrotic regions

		HIF-1α^+^/Pimo^−^	Pimo^+^/HIF-1α^−^	
pH	Neutral (if WE is low)	Acidic (pH ≥ 6.91)	Acidic (pH ≥ 6.91)	Acidic (mean pH = 6.70)
	Acidic (if WE is high)			

WE: Warburg effect.

**Table 5:** Proliferative Activity of Cancer Cells in Each Layer of a Micro Tumor Cord.

	Normoxic regions	Hypoxic regions		Necrotic regions

		HIF-1α^+^/Pimo^−^	Pimo^+^/HIF-1α^−^	
Proliferative Activity & Cell Cycle Status	Highly Proliferative	Controversial (G_1_ Arrest or Still Proliferative)	Arrested at S Phase (Substantially G_1_/G_0_)	−

**Table 6:** Radioresistance of Cancer Cells in Each Layer of a Micro Tumor Cord.

	Normoxic regions	Hypoxic regions		Necrotic regions

		HIF-1α^+^/Pimo^−^	Pimo^+^/HIF-1α^−^	
Radioresistance	Extremely Low	Low	High	−
Contribution to Recurrence	−	−/+	+++	−
